# *Streptococcus pyogenes* endometritis- a population-based study on the causative *emm*-types, case clustering and disease severity

**DOI:** 10.1007/s10096-026-05491-8

**Published:** 2026-04-01

**Authors:** Anja Carblom, Erik Senneby, Omar Sigurvin Gunnarsson, Ann-Cathrine Petersson, Magnus Rasmussen

**Affiliations:** 1 Department of Infectious Diseases, Skåne University Hospital, Lund, Sweden; 2 Department of Obstetrics and Gynaecology, Hudiksvall Hospital, Region Gävleborg, Hudiksvall, Sweden; 3 Clinical Microbiology, Department of Translational Medicine, Faculty of Medicine, Lund University, Malmö, Sweden; 4 Department of Clinical Microbiology, Infection Control and Prevention, Region Skåne, Lund, Sweden; 5 Department of Obstetrics and Gynaecology, Skåne University Hospital, Lund and Malmö, Sweden; 6 Perinatal and Cardiovascular Epidemiology, Clinical Sciences Malmö, Lund University Diabetes Centre, Lund University, Malmö, Sweden; 7 Department of Clinical Sciences Lund, Division of Infection Medicine, Division of Infection Medicine Diseases, Lund University, BMC B14, Lund, SE-223 63 Sweden

**Keywords:** *Streptococcus pyogenes*, Endometritis, *emm*-type, Outbreak, Post-partum

## Abstract

**Purpose:**

*Streptococcus pyogenes* is a cause of endometritis, a severe infection in women of reproductive age. *S. pyogenes* is classified into *emm*-types based on variations of the *emm*-gene. We aimed to compare the distribution of *emm*-types in endometritis to that of controls with bacteraemia, and to assess associations between *emm*-types and disease severity or clustering of cases.

**Methods:**

Vaginal or cervical cultures of *S. pyogenes* from Departments for gynaecology and obstetrics in Skåne, between 2012 and 2020, were identified. Clinical data on patients were retrieved from medical records. Inclusion required an event (childbirth, abortion, miscarriage, intrauterine device insertion or gynaecologic surgery) within 42 days prior to symptom onset. A cluster was defined as *≥* 2 patients with the same *emm*-type at the same hospital within 14 days. *Emm*-types of isolates from post-event infections were compared to those of isolates from blood.

**Results:**

A total of 120 patients were identified, 107 had endometritis and 13 had post-event fever. Vaginal delivery was the most common preceding event (79%). Twenty-six patients (22%) fulfilled sepsis criteria, 15 patients required surgical intervention and six were admitted to the ICU. The distribution of *emm*-types differed significantly between groups (*p* < 0.001): *emm89* was most prevalent in endometritis (21%), whereas *emm1* predominated in bacteraemia (33% of 642 isolates). Five suspected clusters involving 12 patients were identified. No significant associations were found between *emm*-type and disease severity or clustering.

**Conclusion:**

The *emm*-type distribution differed between endometritis and bacteraemia groups, suggesting variation in the propensity of specific *emm*-types to cause endometritis.

## Introduction

*Streptococcus pyogenes* is the leading cause of severe infection and maternal mortality in the postpartum period, with endometritis being the most common underlying condition [1, 2]. The risk of acquiring an invasive *S. pyogenes* infection is estimated to be 20-fold higher in women postpartum, compared to non-pregnant women [3]. A systematic review conducted in 2023 identified a total of 1160 patients with pregnancy and puerperal *S. pyogenes* infection, where bacteraemia accounted for 49% and endometritis for 46% of cases [4]. Of the 1160 patients, 28% developed puerperal sepsis, and the overall case fatality was 2% [4].

The Centers for Disease Control and Prevention (CDC), suggests that endometritis should be diagnosed in patients with suspected infection who present with at least two of the following signs or symptoms: fever (> 38.0 °C), uterine tenderness, abdominal pain, or purulent discharge, in the absence of another recognized cause [5, 6].

The underlying factors contributing to increased susceptibility to *S. pyogenes* infection among pregnant and postpartum women remain unresolved [7, 8], although the route of infection may be either exogenous or endogenous [2, 9]. Studies have shown that postpartum infections are more common in women who have undergone caesarean Sect. (7.9%), compared to those who have had a vaginal delivery (1.8%) [10]. Caesarean section has also been reported as the predominant risk factor for postpartum endometritis [11]. In contrast, when reviewing the literature on *S. pyogenes*–induced postpartum infections specifically, the majority of cases are reported following uncomplicated vaginal births [4].

*S. pyogenes* can be classified into different *emm*-types, based on the hypervariable region of the *emm-*gene encoding the amino-terminal part of the M protein [12, 13]. Certain *emm*-types have been associated with specific clinical conditions and outcomes [14]. Historically, *emm*28 has been frequently isolated from patients with postpartum infections [15, 16]. A possible explanation for the propensity of *emm*28 to cause infections of the female genital tract is the expression of the surface protein R28, which mediates bacterial adherence to vaginal epithelial cells [17]. The *emm*28 type is one of the most prevalent types in Sweden [15]. However, other *emm*-types, such as e*mm*1 and *emm*3, have consistently been associated with invasive infections and higher mortality rates than other *emm*-types in several previous studies [18–21].

The yearly incidence of postpartum endometritis in Sweden is not available. However, several countries have reported increases in severe postpartum *S. pyogenes* infections in recent years [22]. The cause is suspected to be multifactorial, with increasing maternal risk factors for infection, as well as predominance of *emm1* and *emm28*, which have been associated with increased morbidity and mortality [22, 23]. Not all patients with *S. pyogenes* endometritis progress to fulminant infection. The objective of this study was therefore to describe the *emm*-type distribution and clinical features of post-event infections in our Region. We also aimed to determine whether specific *emm*-types are associated with an increased propensity to cause postpartum infections, and to assess whether certain *emm*-types occur more frequently in severe disease or in healthcare-associated outbreaks.

## Materials and methods

Patients were identified through searches in the database of the clinical microbiology laboratory in Lund, Sweden. This laboratory serves all healthcare facilities in Region Skåne, a region with approximately 1.4 million inhabitants (Statistics Sweden, 2020 [24]). The study population consisted of all patients with a positive culture of *S. pyogenes* from the vagina or cervix, collected from any of the Departments for Gynaecology and Obstetrics in the region, from February 2012 to March 2020. To be included, an event had to occur have occurred within 42 days prior to the infection. The time-period of 42 days (six weeks) is the period generally referred to as the puerperal period. The events in question were labour, abortion (surgical or medical), miscarriage, gynaecologic surgery, or insertion of an intrauterine device. For labour cases, symptoms could start up to 72 h before delivery. The post-event infections were classified as either endometritis or post-event fever. Endometritis was defined as a positive culture of *S. pyogenes* from the genital tract with at least one of the following: uterine tenderness, abdominal pain or purulent discharge with no other recognized cause [5, 25]. Patients who developed fever with no further localized symptoms of infection (beyond a positive cervical or vaginal culture with *S. pyogenes*) were categorized as post-event fever.

*S. pyogenes* blood culture isolates collected 2012–2020 in Region Skåne served as a control group for *emm*-type comparisons. Patients with bacteremia in conjunction with a post-event infection were excluded from the bacteremia control cohort. Some patients had more than one positive culture for *S. pyogenes* during the same post-event infection episode. Bacterial species determination was performed using Microflex MALDI-TOF MS (Bruker, Bremen, Germany) with the software FlexControl and MBT Compass 4.1 (database MBT-BDAL-6903). The isolates underwent *emm*-typing according to the protocol from US Centers for Disease Control and Prevention [26]. The study was approved by the Swedish Ethical Review Authority, (record number 2020–06278).

The electronic medical records of the study patients were examined by one of the authors (AC) who consulted with the other authors (OSG or MR) in cases of unclarities. The variables included in the analysis were time from event to symptoms, maximum C-reactive protein (CRP) level during hospitalization, days of hospitalization, days of intravenous antibiotic treatment, days of total antibiotic treatment, need of surgery during hospitalization, sepsis, intensive care unit (ICU) admission and death.

### Definitions

*Emm*-types represented by more than five isolates were analysed individually, whereas those with fewer than five isolates were grouped together under the category *others.* A cluster was defined as two or more patients, infected with the same *emm*-type, at the same hospital within a 14-day period. A cluster was considered closed when 14 days had passed since the last case.

Sepsis was defined according to the Sepsis-3 criteria, as an acute increase of ≥ 2 points in the Sequential Organ Failure Assessment (SOFA) score in the presence of suspected or confirmed infection [27]. Peripheral oxygen saturation, platelet count, mean arterial pressure (MAP), Glasgow Coma Scale (GCS) and creatinine were used to evaluate organ function.

### Statistics

Most variables were non-normally distributed, and therefore non-parametric tests were used throughout all analyses. The Chi-Squared test was used when comparing two independent groups with nominal data. For analysis involving ordinal data across more than two independent groups, the Kruskal-Wallis test was employed. Fisher’s exact test was performed when expected frequencies were < 5. IBM SPSS version 27 was used to perform statistical analyses. The significance level was set at a p-value < 0.05 for statistical significance.

## Results

### Study population

**Fig. 1:**
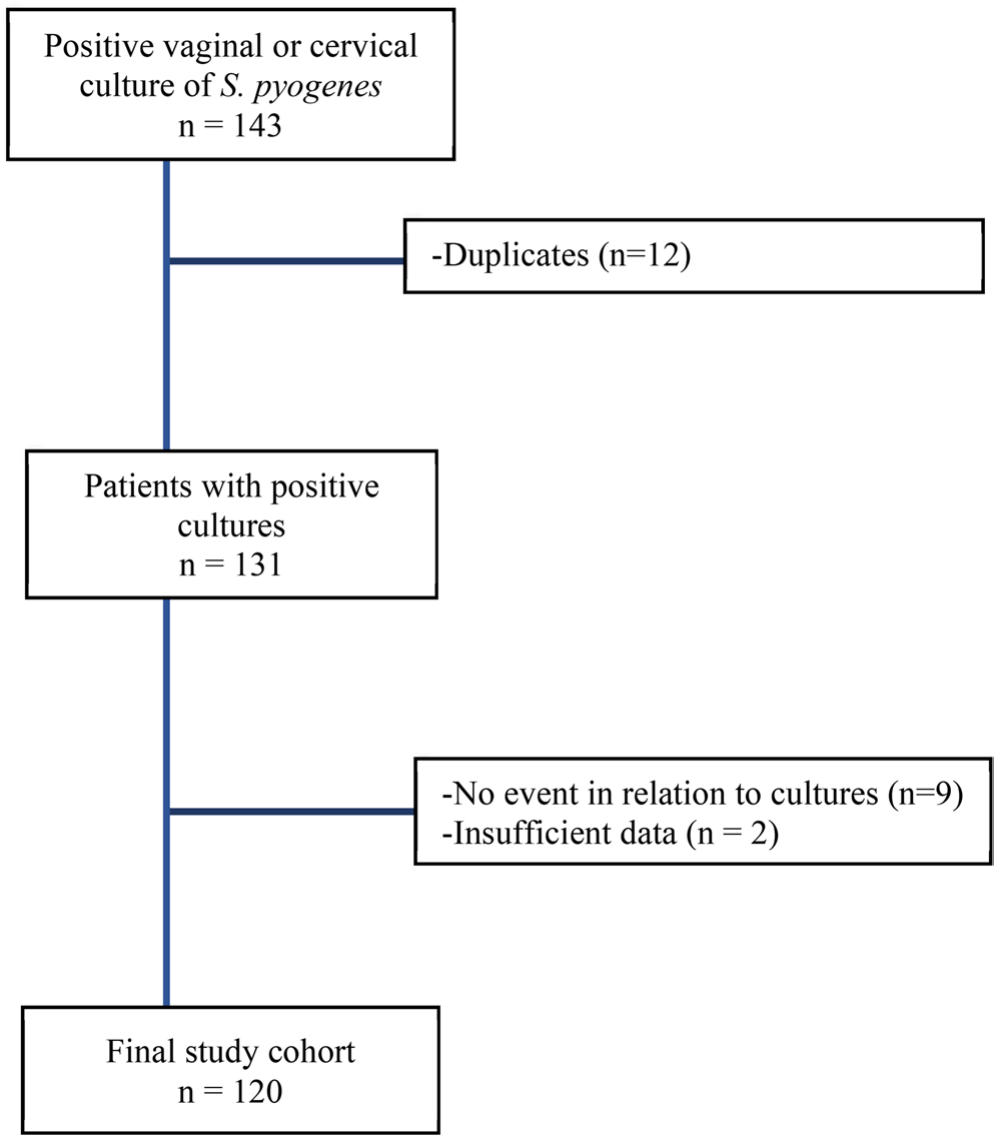
Flow chart of cohort generation

The final study population consisted of 120 patients, of whom 107 had endometritis and 13 patients presented with post-event fever, see Fig. 1 for inclusion and exclusion. In the endometritis group, 32 patients (30%) had a positive blood culture with *S. pyogenes* in addition to a positive cervical or vaginal culture. Patient characteristics in the patient cohort is described in Table 1. The control group comprised 642 patients with bacteraemia after exclusion of the 32 patients with bacteremia and concurrent post-event infection.

**Table 1:** Background information on study cohort, *n* = 120

**Variable**	**Measured data**	
**Endometritis**	107 (89%)	
**Post-event fever**	13 (11%)^1^	
**Age**	32 (28–36)	
**Number of births**	2 (1–2)	
**BMI**^**2**^	24 (21–26)^a^	
**Smoking**	9 (10%)^b^	
**Vaginal childbirth**	95 (79%)	
**Instrumental childbirth**	5 (5.3%)	
**Manual placenta removal**	2 (2.2%)^c^	
**Episiotomy**	6 (6.5%)^d^	
**Elective caesarean section**	0 (0%)	
**Emergency caesarean section**	2 (1.7%)	
**Medical abortion**	5 (4.2%)	
**Surgical abortion**	2 (1.7%)	
**Spontaneous abortion**	2 (1.7%)	
**Insertion of an intrauterine device**	10 (8.3%)	
**Surgery**	4 (3.3%)	
**Antibiotics during labour**	4 (4.2%)^e^	
**Volume of bleeding during labour**	350 (300–500)^f^	
**Week of labour**	40 (38–40)^g^	

Values presented as median (interquartile range) and as an absolute number and percentage of patients, n(%)

^1=^ including one patient with fever developed after abortion (medical followed by surgical)

^2^= calculated at the initial midwife appointment

Missing data: a = 24, b = 30, c = 5, d = 2, e = 1, f = 3, g = 3 patients

### Distribution of *emm*-types

Seven distinct *emm*-types were included in the analysis, and the distribution is presented in Table 2. The *emm*-type category *others* consisted of 11 different *emm*-types identified in the endometritis cohort and 31 types identified in the bacteraemia cohort, which were analysed as common groups. The *emm*-type distribution was significantly different when comparing the 107 endometritis cases to bacteraemia as well as when comparing all 120 post event infections and bacteraemia (*p* < 0.001).

**Table 2:** Distribution of *emm*-types in the study and the control group

emm-type	Post event infection^a^ *n* = 120	Endometritis^a^ *n* = 107	Bacteraemia^a^ *n* = 642
*emm*89	26 (21%)	23 (21%)	74 (12%)
*emm*1	20 (17%)	17 (16%)	212 (33%)
*emm*4	19 (16%)	17 (16%)	43 (7%)
*emm*28	13 (11%)	13 (12%)	89 (14%)
*emm*12	11 (9%)	10 (9%)	49 (8%)
*emm*75	11 (9%)	10 (9%)	14 (2%)
*others*	20^b^ (17%)	17^b^ (16%)	161^c^ (25%)

Values presented as absolute numbers of patients.

^a^ The distribution of *emm-*types is significantly different between the post event infection/endometritis groups and the bacteraemia group (*p* < 0.001 with chi^2^-test)

^b^ *Emm*-types included in others: *emm*3 (*n* = 4), *emm*11 (*n* = 3), *emm*6 (*n* = 3), *emm*2 (*n* = 3), *emm*22 (*n* = 1), *emm*87 (*n* = 1), *emm*5 (*n* = 1), *emm*9 (*n* = 1), *emm*27 (*n* = 1), *emm*44 (*n* = 1), *emm*77 (*n* = 1)

^c^ *Emm-*types included in others: *emm*3 (*n* = 48), *emm*11 (*n* = 5), *emm*6 (*n* = 6), *emm*2 (*n* = 12), *emm*22 (*n* = 7), *emm*87 (*n* = 15), *emm*5 (*n* = 2), *emm*9 (*n* = 4), *emm*27 (*n* = 0), *emm*4 (*n* = 8), *emm*77 (*n* = 7), *emm*102 (*n* = 1), *emm*103 (*n* = 1), *emm*112 (*n* = 2), *emm*118 (*n* = 4), *emm*119 (*n* = 1), *emm*128 (*n* = 1), *emm*140 (*n* = 1), *emm*18 (*n* = 2), *emm*221 (*n* = 1), *emm*232 (*n* = 1), *emm*49 (*n* = 3), *emm*60 (*n* = 1), *emm*73 (*n* = 2), *emm*76 (*n* = 4), *emm*81 (*n* = 3), *emm*82 (*n* = 3), *emm*85 (*n* = 2), *emm*90 (*n* = 2), *emm*94 (*n* = 1), unknown type (*n* = 7)

**Fig. 2:**
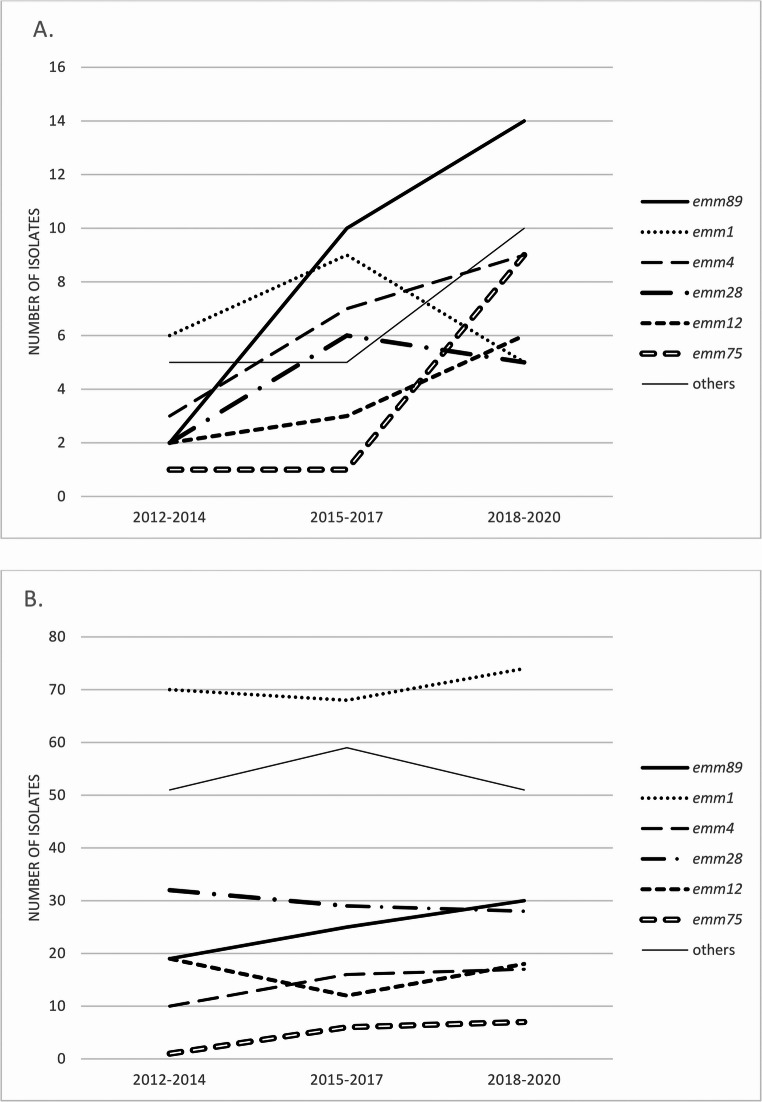
In A. the number of isolates from cervix and vagina of different *emm*-types per time period is shown. B shows the number of bacteremia isolates (after exclusion of women with post-event infection) per time period

In Fig. 2 we present the temporal trends of the different *emm-*types, with time divided into three-year periods, both regarding the post event isolates (Fig. 2A) and bacteremia isolates (Fig. 2B).

### Outcome

The clinical features and outcomes of all patients with post event infection is shown in Table 3. The criteria for sepsis were met in 26 patients (22%).

**Table 3:** Variables concerning the characteristics and severity of all post-event infections

	All isolates *n* = 120	emm89 *n* = 26	emm1 *n* = 20	emm4 *n* = 19	emm28 *n* = 13	emm12 *n* = 11	emm75 *n* = 11	others *n* = 20	*p*-value
Age	32 (28–36)	31 (29–33)	32.5 (30–35)	33 (30–38)	34 (30–38)	34 (27–35)	32 (29–35)	32 (28–36)	0.59^1^
Time from event to symptom onset	2 (1–5)	2.5 (2–5)	2 (1–7.5.5)	2 (1–3.5.5)	3 (1–5)	2 (1–2.5.5)	3 (2–9.5.5)	2 (1–5)	0.78^1^
Maximum CRP during hospitalization	201 (129–289)	168 (117–282)	226 (154–287)^a^	178 (131–247)^b^	331(224–399)^c^	253 (169–290)^d^	238 (165–249)	189 (122–289)	0.10^1^
Days of hospitalization	3 (2–5)	3 (3–5)	3.5 (2–5)	3 (2–5)	4 (2.5–6.5)	4 (2.5–5.5)	4 (3–4.5.5)	3 (2–5)	0.80^1^
Days of intravenous antibiotic treatment	3 (2–5)	3 (2–5)	3 (2–4)	3 (2–4)	5 (2.5–6.5)	3 (2.5–4.5)	3 (2.5–3.5)	3 (2–5)	0.75^1^
Days of total antibiotic treatment	11 (10–13)	11 (10–13)	11 (10–13)^f^	10 (9–13)	13 (11–14)	11 (10–14)	11 (10–13)	11 (10–13)	0.37^1^
Sepsis during hospitalization	26 (22%)	6 (23%)	4 (20%)	2 (11%)	2 (15%)	5 (45%)	4 (36%)	3 (15%)	^2,3^
Surgical intervention	15 (13%)	1 (4%)	3 (15%)	2 (11%)	2 (15%)	4 (36%)	1 (9%)	2 (10%)	^2^
Patients in need of ICU (number of days min-max)	6 (5%)	1 (2)	3 (1–10)	0	0	1 (7)	0	1 (1)	^2^
Clusters	12 (10%)	4 (in 2 clusters)	2	0	0	0	4 (in 1 cluster)	2	^2^
Deaths	1 (0.8%)	0	1	0	0	0	0	0	^2^

Value presented as median (interquartile range) and as an absolute number and percentage of patients, n(%)

Missing information a = 1, b = 1, c = 2, d = 1, e = 2, f = 1 patients

^1^ Kruskal Wallis test comparing all *emm-*groups

^2^ Chi-Squared test was not performed due to expected frequencies < 5

^3^ *emm*12 vs. non-*emm*12 was compared with chi^2^-test (*p* = 0.067)

### Clusters

Five clusters were detected, including a total of 12 patients (Table 3). The cluster of *emm*75 have previously been described [28].

## Discussion

In our study, *emm*89 was the most prevalent type among patients with endometritis (21%). This contrasts to previous reports, identifying *emm*28 as the predominant cause of postpartum endometritis [16]. In our material, *emm*28 accounted for 12% of endometritis cases, while Gröndahl-Yli-Hannuksela et al.. reported that *emm*28 caused endometritis at significantly higher rates than any other *emm*-type [16]. Previous studies have described *emm*28 as one of the dominant *emm*-types in Sweden overall [15], which demonstrates that the *emm-*type distribution in the population varies over time. Notably, *emm*28 was more prevalent in the bacteraemia group as compared to the endometritis group.

There were no significant differences in disease severity or clusters of cases between the different *emm*-types. In line with previous research [18–21], our study suggests that *emm*1 is common in invasive disease. It was the most prevalent type accounting for 33% of the bacteraemia cases, and the second most prevalent type among patients with endometritis. Furthermore, among patients with endometritis who required ICU admission, *emm*1 accounted for 50% of cases and was present in the only fatal case.

A key strength of this study is the use of a population-based cohort with a microbiologically well-defined study group. Routine *emm*-typing of isolates at our laboratory ensures high data completeness and supports the robustness of the findings. A limitation of this study is the retrospective design, which restricts data collection to information documented in the patient charts. A prospective study would be preferable to ensure more comprehensive and standardized data collection, however, the relatively low incidence of *S.* p*yogenes* infections makes it challenging to gather enough cases within a reasonable timeframe. Additionally, the focus on *S. pyogenes* may be considered a limitation since endometritis can be caused by a variety of other pathogens including *Streptococcus dysgalactiae* [25].

There is a lack of a universal definition of endometritis, which makes comparison of different studies and populations difficult. We used a definition where local symptoms together with *S. pyogenes* in a vaginal or cervical culture was needed. For 13 patients with fever, no distinct focal symptoms were noted.

A total of five clusters involving twelve patients were identified, in which nosocomial transmission can be suspected. However, we do not know if these clusters are caused by nosocomial transmission since a cluster is only defined in terms of *emm*-type and temporal and spatial relationship. Our definition of a cluster risk to underestimate true outbreaks by missing nosocomial transmission with more than 14 days between cases. For example, only four of the five cases in the outbreak previously described by Trell et al. [28] were identified since the last case of that outbreak case occurred more than 14 days after the preceding case. Our definition can also overestimate the number of outbreaks since different clones of *S. pyogenes* bacteria might carry the same *emm-*gene. This is also exemplified in the outbreak described by Trell et al. [28] where a sixth case of *S. pyogenes* endometritis caused by the same *emm-*type within the same time period was found to be clonally unrelated to the five isolates which were nosocomially transmitted.

Previous studies have identified caesarean section as an important risk factor for endometritis [10, 11]. However, in our cohort, no patients underwent elective caesarean section, and only two cases followed acute caesarean section. With our study design, we were unable to determine risk factors for postpartum endometritis, as we did not have a control group with non-endometritis patients for comparison.

In conclusion, the *emm*-type distribution differed significantly between patients with endometritis and those with bacteraemia in the general population, suggesting that certain *emm*-types may have an increased propensity to cause endometritis.

## Data Availability

Pseudonymized data will be available upon reasonable request.
